# Development and evaluation of optimized PCR and indirect ELISA for the detection of *Morganella morganii* in dairy cows

**DOI:** 10.3389/fvets.2025.1532600

**Published:** 2025-01-15

**Authors:** Meihua Zhang, Jiayi Li, Jianfeng Xue, Huiling Xu, Muzi Li, Yibo Xia, Changxi Qi, Pu Zhang, Yongxia Liu, Jianzhu Liu

**Affiliations:** ^1^College of Veterinary Medicine, Shandong Agricultural University, Tai’an, China; ^2^Shandong Provincial Key Laboratory of Zoonoses, Shandong Agricultural University, Tai’an, China; ^3^The Affiliated Tai’an City Central Hospital of Qingdao University, Tai’an, China

**Keywords:** enzyme linked immunosorbent assay, epidemiological investigation, lipoprotein, *Morganella morganii*, polymerase chain reaction

## Abstract

**Introduction:**

*Morganella morganii* (*M. morganii*) is a Gram-negative opportunistic pathogen, whose increasing virulence and antibiotic resistance negatively impact dairy cow health and productivity, raising concerns in livestock health management. To mitigate this risk, rapid and reliable diagnostic methods for detection are essential. Currently, detection methods for *M. morganii* are underdeveloped, prompting us to develop both pathogenic and serological detection methods, including an optimized PCR technique and an indirect enzyme-linked immunosorbent assay (I-ELISA).

**Methods:**

The optimized PCR method utilized bacterial suspensions directly as templates, bypassing the need for DNA extraction and thereby allowing the direct detection of M. morganii in fecal samples. Primer concentrations and annealing temperatures were optimized to minimize primer dimer formation, ensuring high specificity. Clinical evaluation was conducted using 771 fecal and nasal fluid samples collected from dairy farms in five regions. The I-ELISA method was developed using *M. morganii* lipoprotein (LPP) antigen. Parameters such as antigen coating, blocking conditions, and antibody dilution were optimized to improve specificity. Stability and reproducibility were validated through intra- and inter-assay tests. A total of 476 serum samples from dairy cows were tested to assess the method’s clinical applicability.

**Results:**

The optimized PCR method demonstrated high sensitivity and specificity, achieving a detection threshold of 0.2 CFU/μL. Clinical testing revealed a positivity rate of 1.4% among 771 fecal and nasal fluid samples. The I-ELISA method showed excellent stability and reproducibility, confirmed through intra- and inter-assay consistency. In testing 476 dairy cow serum samples, the positivity rate for *M. morganii* was 5.9%. These results indicate the utility of I-ELISA as a reliable serological diagnostic tool.

**Discussion:**

The PCR and I-ELISA methods collectively offer practical solutions for the early clinical diagnosis of *M. morganii* infections in dairy cows. The PCR technique’s efficiency and sensitivity make it ideal for pathogen detection in fecal samples, while the I-ELISA method provides a robust platform for serological analysis. Together, these tools enable timely intervention, contributing to improved livestock health management and mitigating the negative impacts of *M. morganii* on dairy cow productivity. Future research may focus on further refining these techniques and exploring their applications in broader livestock management contexts.

## 1 Introduction

*Morganella morganii*, a species belonging to the genus *Morganella* within the Enterobacteriaceae family, was first identified in the 1990s and is widely distributed in nature, particularly in the intestines of animals like cattle and pigs (1–4). As an opportunistic pathogen, *M. morganii* can cause severe diseases, such as sepsis, abscesses, urinary tract infections, cellulitis, diarrhea, and bacteremia, especially when the host’s immune defenses are compromised or favorable environmental conditions arise (5–8). Its pathogenicity is closely linked to endotoxins and the organism’s virulence factors. Endotoxins trigger complex immune responses in the host, while virulence factors determine the degree of harm inflicted on the host (9, 10). *M. morganii* secretes hemolysins and features multiple flagella. Hemolysins damage host tissues and cells, while the multiple flagella enhance bacterial motility and spread within the host, collectively impacting respiratory health and leading to decreased immunity. In modern animal husbandry, the long-term use of broad-spectrum antibiotics disrupts normal intestinal flora, creating favorable conditions for *M. morganii* growth, which can proliferate rapidly and cause disease when host immunity is compromised.

In recent years, cases of *M. morganii* infection have been on the rise, garnering significant attention. Studies have shown that *M. morganii* infections can be fatal in pigs, with isolates from the intestinal tracts of deceased pigs causing varying degrees of hemorrhage in the liver, intestines, and lungs of mice, with a mortality rate as high as 60% (11). In August 2011, multiple batches of one-day-old broiler chicks from a hatchery in Tianjin, China, were infected with *M. morganii*, exhibiting high mortality rates along with characteristic lesions such as diffuse hepatic hemorrhage and pulmonary edema (12). In 2014, an outbreak of *M. morganii* infection occurred at a Chinese giant salamander farm in Shanxi Province. Affected salamanders displayed symptoms including lethargy and abdominal distension. Post-mortem examinations revealed abdominal effusion, hepatic hemorrhage, and hyperemia of intestinal capillaries, with a mortality rate similarly reaching 60% (13). These cases underscore the highly pathogenic nature of *M. morganii* infections and their serious threat to livestock, poultry, and aquatic animals. Likewise, *M. morganii* poses a significant risk to the cattle industry. In 2017, an outbreak at a cattle farm in Shandong, China, revealed severe health issues among neonatal calves infected with *M. morganii*, including depression, paralysis, and gastrointestinal symptoms, with a mortality rate reaching 57%. Surviving calves exhibited stunted growth and emaciation. Despite treatment with various antibiotics, the lack of efficacy highlighted the bacterium’s resistance to commonly used drugs (14). A separate study conducted in Dhaka, Bangladesh, analyzed the genomic characteristics of three *M. morganii* strains isolated from bovine rectal swabs. Genomic analysis demonstrated that *M. morganii* harbors a wide array of antibiotic resistance genes (e.g., *blaCTX-M*, *blaTEM*) and virulence factors (e.g., hemolysins, cytotoxins), which not only enhance its pathogenic potential but also complicate disease management (15). Furthermore, its genetic adaptability to environmental conditions increases the risk of dissemination within farms, posing severe threats to cattle health and productivity while also raising concerns about food safety and public health. In China’s economy, the dairy industry is pivotal, and cattle health directly affects milk yield and quality, subsequently impacting the economic viability of the dairy sector. Thus, establishing an efficient detection method to monitor and control *M. morganii* infections in cattle is crucial.

PCR and ELISA are widely used for pathogen detection due to their high sensitivity, specificity, and rapid, reliable results (16, 17). PCR can detect pathogens at low concentrations, making it suitable for early diagnosis, and quantitative PCR (qPCR) can provide pathogen load data useful for disease evaluation and monitoring. ELISA, being straightforward to perform, is suited to large-scale screening, offering stable reagents, low equipment costs, and minimal environmental requirements. Additionally, ELISA leverages the specificity of antigen–antibody interactions to accurately assess infection stages and immune responses. In comparison, other methods face limitations: culture methods are time-consuming and require stringent conditions; immunochromatographic assays are less sensitive and specific; microscopy lacks the resolution to detect low pathogen concentrations; and nucleic acid hybridization is complex and costly (18–20). Therefore, PCR and ELISA provide a balanced advantage of sensitivity, specificity, and convenience, making them ideal for rapid detection and epidemiological screening.

*M. morganii* lipoprotein (LPP) represents a lipid-modified membrane protein that occupies a central position in a multitude of physiological processes, including cell membrane synthesis, nutrient absorption, cell division regulation, and transmembrane signal transduction (21–24). Moreover, LPP may participate in antibiotic resistance mechanisms, allowing *M. morganii* to survive in antibiotic-rich environments. The DNA sequence of the LPP gene has been determined, and its highly conserved structure highlights its critical role in the bacterium’s survival and proliferation, where it acts as a virulence factor directly involved in multiple pathogenic mechanisms, posing a threat to host health (25).

Given LPP’s essential role in *M. morganii*, its conserved structure, and strong association with virulence, targeting LPP for PCR and indirect ELISA diagnostics is highly promising. Developing specific detection methods targeting LPP could enable rapid detection of *M. morganii*, providing essential technical support for infection monitoring and control, ensuring animal health, and enhancing the economic benefits of livestock industries.

In this study, we thoroughly investigated a direct liquid PCR detection method for *M. morganii*, introducing a direct PCR technique that resolves challenges associated with bacterial liquid PCR, providing a novel approach for rapid detection. Additionally, we developed an indirect ELISA diagnostic method based on *M. morganii* antigen protein LPP. By employing gene cloning and protein purification, we established a specific and stable ELISA method that effectively distinguishes anti-*M. morganii* antibodies from cross-reactive antibodies against other pathogens. The optimized PCR technique is primarily used to detect bacterial nucleic acids, exhibiting high specificity for bacterial DNA in samples. It is suitable for early infection detection, including fecal and tissue samples, especially in cases of low bacterial load. In contrast, I-ELISA mainly targets antibodies in serum and is more suitable for large-scale serological surveys to assess the infection history and immune status of cattle herds. The selectivity of these two detection methods varies depending on the sample type, with each having its own advantages in different scenarios of disease monitoring and control.

## 2 Materials and methods

### 2.1 Materials

The strains *M. morganii* SDTA1, SDTA2, SDTA3, SDTA4, and SDTA5 are isolated from the small intestine, liver, spleen, lung, and kidney of calves, respectively. *M. morganii* strains are purchased from the BeNa Culture Collection, while strains of *Salmonella*, *Bacillus subtilis*, *Staphylococcus aureus*, *Escherichia coli*, and *Pasteurella* are maintained in our laboratory. DNA markers, dNTPs, Taq DNA polymerase, ddH₂O, DNA extraction kits, etc., are purchased from GenStar Biotechnology Co., Ltd. Primers are synthesized by Shenggong Bioengineering (Shanghai) Co., Ltd. DMEM/high glucose and Trypsin-EDTA solutions are purchased from Gibco (United States). Hoechst 33342 is obtained from the Beyotime Institute of Biotechnology (Haimen, China). All bacteria and 293 T cells used in this study are maintained in our laboratory. Fetal bovine serum is sourced from Biolnd (United States), and penicillin and streptomycin are obtained from Solarbio (Beijing, China). Primary antibodies against His tags are purchased from Abcam (United States). The secondary antibody, goat anti-mouse IgG (H + L) (SA00001-1), is obtained from Proteintech (Chicago, United States), and the His-tagged protein purification kit is acquired from GenScript (Nanjing, China).

### 2.2 Isolation and identification of *Morganella morganii*

Prepare LB liquid medium, autoclave it, and allow it to cool on a clean bench before use. Use a pipette to extract a portion of the bacterial solution and add it to the LB medium, mixing well. Incubate on a shaker at 37°C and 220 rpm for 12 h. Prepare standard solid nutrient agar, autoclave it, and, when it cools to 50–60°C, pour it into petri dishes. Once solidified, inoculate with bacteria. Dip a sterile inoculation needle into the bacterial solution, streak-inoculate onto a solid nutrient agar plate, and incubate for 8–12 h in a 37°C incubator. To obtain pure cultures, pick a single colony for Gram staining and observe it under a microscope. Perform 16S rRNA and biochemical identification for further confirmation. Use both isolated strains and commercial strains in subsequent experiments to ensure the accuracy and applicability of the construction method.

### 2.3 Primer design

A pair of specific primers is designed using NCBI based on the conserved sequence of the antigen protein gene of *M. morganii* (registration number MU9_RS00145) to determine the target sequence for primer design. Several conserved regions are selected for this purpose. The forward primer is 5′-ATGCATTATGAT ACCCATCAGA-3′, and the reverse primer is 5′-TCATTCACCCTT ATGAAAGA-3′.

### 2.4 DNA extraction and PCR amplification

This experiment employs two methods for DNA extraction. One method uses a DNA kit to extract total DNA from *M. morganii* by centrifuging a 2 mL bacterial solution at 10,000 rpm for 2 min, yielding an elution volume of 200 μL. The second method involves direct amplification from the bacterial liquid, where a 2 mL volume is centrifuged at 10,000 rpm for 2 min, the supernatant is discarded, and the pellet is resuspended in sterile PBS solution. This washing step is repeated three times before proceeding with PCR.

### 2.5 Establishment of PCR program

The PCR reaction system consisted of a total volume of 25 μL, including 5 μL of bacterial suspension, 1 μL of forward primer, 1 μL of reverse primer, and 18 μL of ddH₂O. To determine the optimal primer concentration, a series of gradient concentrations were tested, specifically 0.1 μM, 0.2 μM, 0.3 μM, and 0.5 μM. Additionally, the annealing temperature was optimized using a gradient PCR approach, with tests performed at 45°C, 50°C, 55°C, and 60°C. The remaining PCR amplification conditions were as follows: initial denaturation at 94°C for 5 min; denaturation at 94°C for 30 s; extension at 72°C for 1 min and 30 s, repeated for 32 cycles; and a final extension at 72°C for 10 min.

### 2.6 Specific detection

This test method is used to perform specificity testing, selecting *M. morganii*, *Salmonella*, *Bacillus subtilis*, *Staphylococcus aureus*, *Escherichia coli*, and *Pasteurella* as test organisms. The PCR amplification is performed using this method, followed by detection through agarose gel electrophoresis.

### 2.7 Repeatability test

Conduct PCR repeatability experiments to verify the efficiency of the primers and the consistency of the amplification products. The experiments utilize template bacterial solutions of the same concentration, primers with identical concentration and volume, and the same batch of PCR reagents. Under consistent PCR amplification conditions, the amplification products are analyzed using agarose gel electrophoresis to observe the consistency of band intensity and position. Each group of experiments is repeated 3 times to evaluate the stability of the results.

### 2.8 Sensitivity detection

A single colony of *M. morganii* is inoculated into broth medium, mixed well, and cultivated overnight at 37°C until the culture becomes cloudy, resulting in the original bacterial solution. One milliliter of this bacterial stock solution is added to a test tube containing 9 mL of sterile water and mixed thoroughly to create a 10^−1^ dilution. This process is then repeated for subsequent dilutions. Each dilution is subjected to PCR and agarose gel electrophoresis for detection. Additionally, each dilution is tested in 3–5 replicates, inoculated into culture medium, and colony counts are performed.

### 2.9 Clinical application

Dilute 10 stool samples confirmed to contain *M. morganii* and 10 stool samples confirmed not to contain *M. morganii* with sterile water at a dilution of 1:8. Filter out residues using a 0.45 μm filter, then centrifuge the filtrate at 3,000 rpm for 5 min. Discard the supernatant and resuspend the precipitate in sterile PBS buffer to obtain a bacterial suspension with an optical density (OD) of 0.1. PCR amplification is then performed using this experimental kit, and the PCR products are analyzed by agarose gel electrophoresis. It is found that all stool samples confirmed to contain *M. morganii* produced an amplified band at 857 bp, while the stool samples confirmed not to contain *M. morganii* did not show amplification at this size, demonstrating consistency with actual results.

From December 2019 to December 2020, fecal and nasal fluid samples from adult cattle and calves were collected at dairy farms in five provinces, including Shandong, Zhejiang, Jiangxi, Anhui, and Heilongjiang. Stool samples were diluted with sterile water, filtered through a 0.45 μm filter, centrifuged at 3,000 rpm for 5 min, and the supernatant was discarded before resuspending the precipitate for PCR.

### 2.10 Construction of recombinant plasmids

Bacterial DNA is extracted from *M. morganii* using a bacterial DNA kit, and the purified DNA is used as a template for PCR amplification. A product encoding the LPP protein, consisting of 90 amino acids, is generated using the forward primer 5′-GCTAGCGCC CCACCATG-3′, which contains an Nhe I restriction site (underlined), and the reverse primer 5′-GAATTCTCAGTGATGGTG GTGGTG-3′, which includes a stop codon and an EcoR I restriction site (underlined). The PCR amplification is conducted under the following cycling conditions: 94°C for 5 min (initial denaturation), followed by 32 cycles of 94°C for 0.5 min (denaturation), 50°C for 0.5 min (annealing), and 72°C for 1.5 min (extension), with a final extension step at 72°C for 10 min. The 276 bp product generated is confirmed to be correct for this gene by DNA sequencing. The insert fragment is obtained from the 276 bp product by digestion with Nhe I and EcoR I and is ligated into the pcDNA3.1(+) expression vector, which is then used to transform the *E. coli* DH5α strain. The construction of the final selected expression plasmid, pcDNA3.1(+)-LPP, is confirmed by DNA sequencing. The pcDNA3.1(+)-LPP plasmid is subsequently transformed into 293 T cells for expression.

### 2.11 Protein expression of recombinant plasmids

The cells were cultured in DMEM medium containing 10% fetal bovine serum and antibiotics at 37°C in a 5% CO₂ atmosphere. Subsequently, 0.01 μg/μL of NANO 293 T was added to induce protein expression. The samples were divided into different groups, all subjected to the same treatment conditions, and incubated for 0, 24, 48, and 72 h, respectively. Subsequently, His-tag antibodies and fluorescent secondary antibodies were used to label the LPP protein. Finally, fluorescence microscopy was employed to observe protein expression at different time points. The expressed LPP protein, collected after growth under optimized conditions, is tested for solubility using the lysis method according to the manufacturer’s instructions. The insoluble protein in the cell debris and the soluble protein in the supernatant are analyzed by electrophoresis through 12% SDS-PAGE gels, followed by western blotting analysis using an anti-His monoclonal antibody as the primary antibody and horseradish peroxidase-conjugated goat anti-mouse IgG as the secondary antibody. Soluble LPP protein from 25 mL of cell culture is purified using Ni-NTA affinity chromatography and anionic denaturing detergents according to the manufacturer’s manual.

Briefly, cell pellets are lysed by adding RIPA lysis buffer at a ratio of 1:20 (RIPA lysis buffer volume: cell culture volume), and the cell lysates are incubated on ice at 4°C for 15 min. Cell debris is removed from the supernatants by centrifugation at 12,000 × g for 10 min at 4°C. The supernatant is adjusted to 8 mL with LE Buffer and passed through 0.45 μm filters before loading onto a Ni-NTA affinity column that has been equilibrated with LE Buffer. The unbound proteins are washed away using an elution buffer. The His-tagged LPP protein is then eluted by adding elution buffer containing imidazole. Finally, any remaining proteins are stripped from the column using a stripping buffer. Each fraction is collected and analyzed by electrophoresis through 12% SDS-PAGE gels and western blotting analysis using an anti-His monoclonal antibody as the primary antibody and horseradish peroxidase-conjugated goat anti-mouse IgG as the secondary antibody. The eluted fractions of purified protein are pooled and concentrated. The concentration of the dialyzed protein is determined using the Bradford assay with bovine serum albumin (BSA) as a standard.

### 2.12 Sera preparation by immunization with *Morganella morganii* and *Morganella morganii* whole protein

Twenty SPF mice (aged 6–8 weeks and weighing 17–23 g each) are immunized via the abdominal cavity with 50 μg of *M. morganii* and *M. morganii* whole proteins emulsified in an equal volume of Freund’s complete adjuvant (FCA). Subsequently, two booster doses of 100 μg of antigen emulsified in an equal volume of Freund’s incomplete adjuvant (FIA) are injected subcutaneously on days 14 and 28. Serum samples are then collected from the immunized mice. Pre-immunization (day 0) serum is considered as negative serum. All collected serum samples are used in western blot analysis and optimization of the LPP-based indirect ELISA.

### 2.13 Antigenicity testing of the LPP recombinant protein

Purified LPP protein is subjected to electrophoresis through 12% SDS-PAGE gels, and the protein is transferred onto polyvinylidene difluoride (PVDF) membranes using a transfer cell at 75 V for 55 min. The membranes are blocked by incubation with 5% skim milk in PBST and then probed with a 1:500 dilution of a polyclonal antibody kindly provided by the lab. The membranes are subsequently incubated with a 1:4,000 dilution of horseradish peroxidase-conjugated goat anti-mouse IgG. The antibody-antigen complex bands are visualized by chemiluminescence using the detection system.

### 2.14 Establishment of LPP indirect ELISA

The LPP indirect ELISA is standardized using checkerboard titration. The maximal signal-to-noise (S/N) ratio (OD_450_ nm of positive serum/OD_450_ nm of negative serum) is determined to select the optimal amount of antigen and dilution of serum and conjugate for the indirect LPP ELISA. Briefly, wells of a 96-well ELISA plate are loaded with 100 μL of LPP (3.2, 1.6, 0.8, 0.4, 0.2, 0.1, 0.005, or 0.0025 μg LPP per well) in coating buffer (deionized water, carbonate solution, phosphate solution, pH 9.6) and incubated at 2–8°C overnight (37°C for 1 h, followed by 37°C for 2 h). The wells are washed with 200 μL PBST three times and then blocked with 200 μL of blocking solution (1, 3%, or 5% skim milk in PBST; 1, 3%, or 5% BSA) and incubated at room temperature for 1 h (37°C for 2 h, 4°C for 12 h). Test sera, including *M. morganii* positive and *M. morganii* negative sera, are diluted (1:100 to 1:204,800) in blocking buffer on a dilution plate, and 100 μL is added to each well of the ELISA plate. After incubation at room temperature for 1 h (30, 45, 60, or 75 min), the wells are washed with 200 μL PBST three times. Then, 100 μL of horseradish peroxidase-conjugated rabbit anti-mouse IgG (H + L), diluted (1:5,000, 1:10,000, 1:20,000, 1:40,000, and 1:80,000) in blocking solution, is added to each well, and the plate is incubated at room temperature for 1 h (30, 45, 60, or 75 min). Wells are washed with 200 μL PBST three times, and the color is subsequently developed by adding 100 μL of 3,3′,5,5′-tetramethyl-benzidine (TMB) and incubating at room temperature for 40 min. The reaction is terminated by the addition of 100 μL stop solution (2 M H_2_SO_4_) per well and incubated for 5 min before the absorbance (OD_450_ nm) is read using a microplate reader.

### 2.15 Cutoff value determination of LPP indirect ELISA

A total of 36 sera, known to be negative after screening, are evaluated using our LPP indirect ELISA test. The cutoff value for positive sera is calculated as (OD_450_ nm of the average serum value +3 × OD_450_ nm of the serum standard deviation). The cutoff value for negative sera is calculated as (OD_450_ nm of the average serum value +2 × OD_450_ nm of the serum standard deviation). Positive and negative control sera are tested on all plates, and all samples, including control sera, are assessed. Samples with values higher than the optimal cutoff value are considered positive, while those equal to or lower than the optimal cutoff are considered negative.

### 2.16 Evaluation of LPP-based I-ELISA

To rule out cross-reactivity of the LPP protein with other related bacteria in cows, serum samples from cows infected with *Salmonella*, *Bacillus subtilis*, *Staphylococcus aureus*, *Escherichia coli*, and *Pasteurella* are used in I-ELISA with the LPP protein. Furthermore, to assess the diagnostic potential of the recombinant protein, a total of 24 serum samples, known to be negative/positive after screening, are evaluated using our LPP indirect ELISA test. Positive and negative control sera are tested on all plates (within and between batches), and all samples, including control sera, are tested three times. The coefficient of variation is calculated as:


$$CV\;\left(\%\right)=\frac{SD}{\bar{X}}\times 100$$

### 2.17 Concordance experiment

To verify the accuracy of the established indirect ELISA detection method for the LPP antibody, we selected 50 bovine serum samples stored in the laboratory. These samples have been previously identified for *M. morganii* infection status through pathogen isolation, identification, and PCR analysis. The samples were tested using the indirect ELISA method, and the results are comparatively analyzed to calculate the concordance rate of the ELISA detection method.

### 2.18 Clinical applications

Random field serum samples (*n* = 476) were collected from cows that visited pastures in Jining, Yantai, Weifang, Zaozhuang, Tai’an, Linyi, Dezhou, Dongying, Liaocheng, Zibo, Heze, and Jinan in Shandong Province. These samples were used to evaluate the LPP-based I-ELISA.

### 2.19 Statistical analysis

Statistical analyses were performed using SPSS software (SPSS18.0). All values given in the text are the mean ± SD from the experiment.

## 3 Results of PCR

### 3.1 Optimization of amplification conditions and successful expression of the target gene

**Figure 1:**
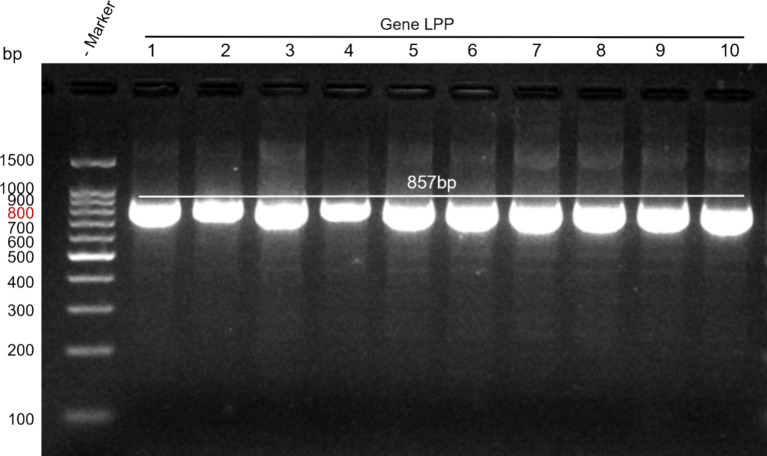
Amplification results of the target gene. Lane M: DNA marker; Lanes 1–10: *M. morganii* strains.

Primer concentration plays a critical role in the efficiency and specificity of PCR amplification. At a concentration of 0.1 μM, the amplification efficiency was low, and the target bands were weak. At 0.2 μM, the target bands were clear, and nonspecific amplification was minimized. At concentrations of 0.3 μM and 0.5 μM, there was no significant improvement in the intensity of the target bands, but nonspecific bands increased. Therefore, a primer concentration of 0.2 μM was determined to be optimal, providing the best amplification performance and specificity, and was selected as the final concentration. The annealing temperature is another key factor influencing the specificity of primer-template binding. A gradient PCR approach was used to optimize the annealing temperature. At 45°C, the amplification yielded more products but showed prominent nonspecific bands. At 50°C, the target bands were moderately intense with good specificity and minimal background noise. At 55°C and 60°C, the amplification efficiency decreased, and the intensity of the target bands weakened significantly. Thus, 50°C was chosen as the optimal annealing temperature. Using the designed primers and optimized amplification conditions, an 857 bp band was successfully amplified, consistent with the expected size and demonstrating good repeatability (see Figure 1).

### 3.2 Evaluation of PCR template selection

**Figure 2:**
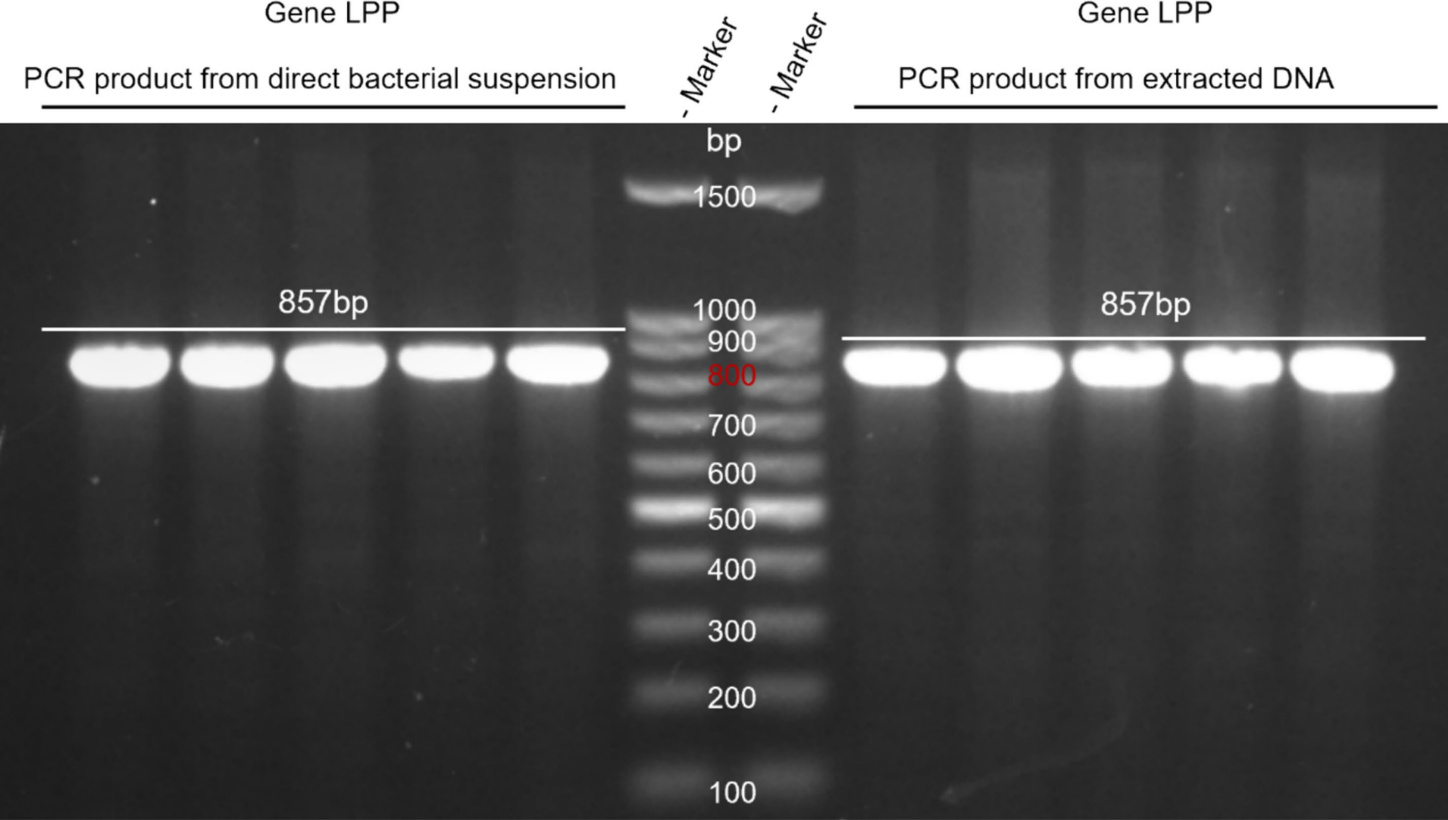
Comparison of DNA extraction and direct PCR methods. Lane M: DNA marker; Left lane: PCR product from direct bacterial suspension. Right lane: PCR product from extracted DNA.

DNA from *M. morganii* was extracted, and direct PCR from the bacterial suspension was performed. The products obtained were analyzed by electrophoresis. Results indicated that both methods successfully yielded the target product, with the direct amplification method proving to be more convenient and recommended as the preferred approach (see Figure 2).

### 3.3 Validation of PCR specificity for target gene detection

**Figure 3:**
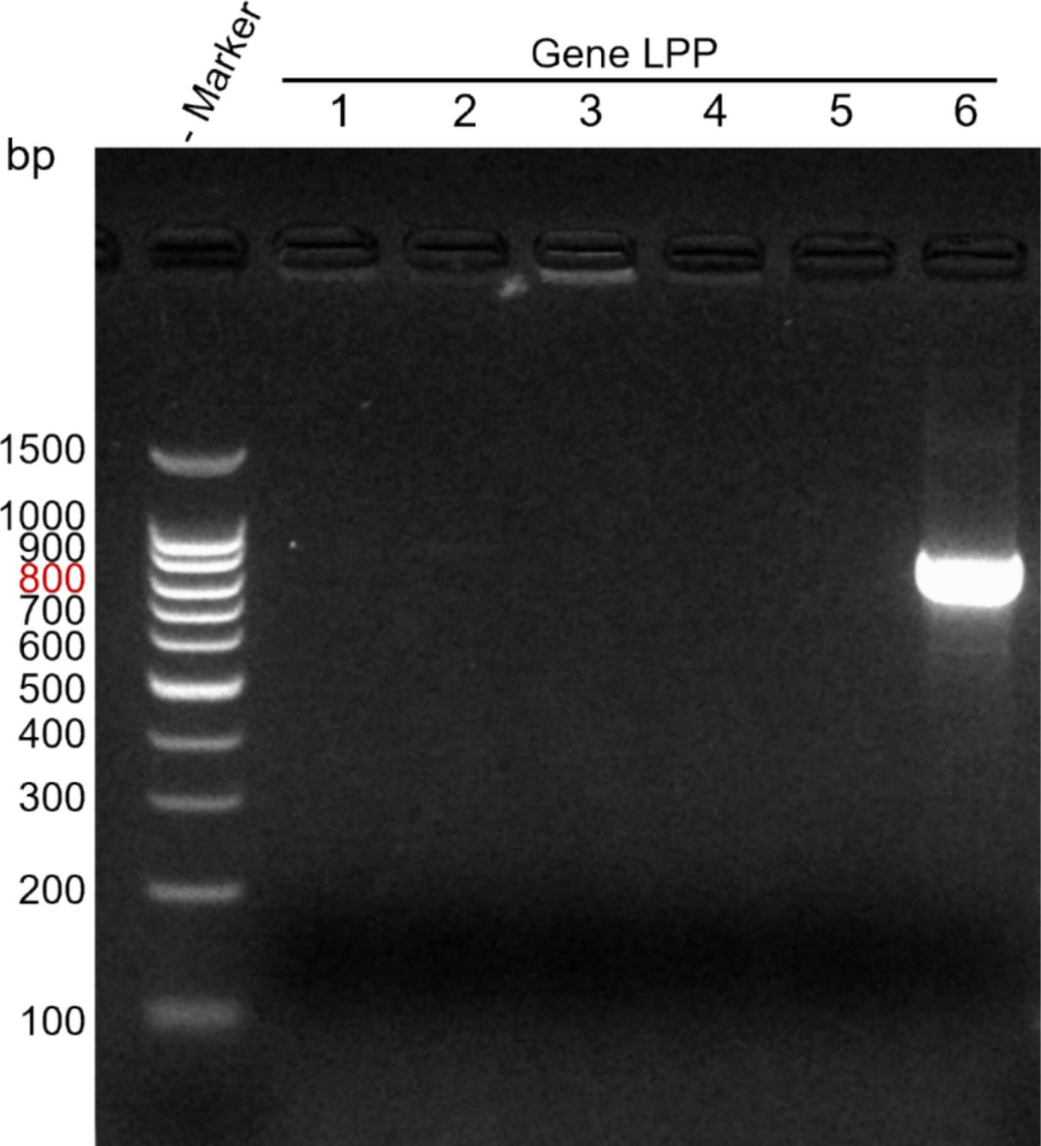
Specificity detection of direct PCR method. Lane M: DNA marker; Lanes 1–6: *Salmonella*, *Bacillus subtilis*, *Staphylococcus aureus*, *Escherichia coli*, *Pasteurella*, *Morganella morganii*.

Using *M. morganii*-specific primers, PCR was conducted on *M. morganii*, *Salmonella*, *Bacillus subtilis*, *Staphylococcus aureus*, *Escherichia coli*, and *Pasteurella*. Subsequent SDS-PAGE electrophoresis revealed that only *M. morganii* produced a target band, confirming the specificity of this PCR method (see Figure 3).

### 3.4 Sensitivity analysis of PCR assays

**Figure 4:**
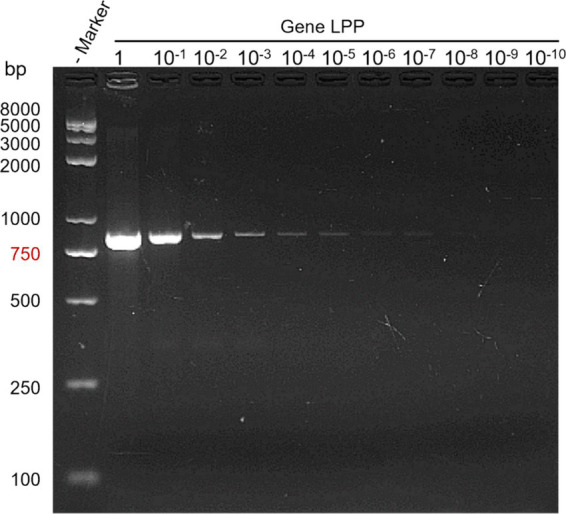
PCR sensitivity test results. Lane M: DNA marker; Lane 1: bacterial stock solution; Lanes 10^−1^ to 10^−10^: dilution factors.

Bacterial stock solutions were subjected to serial dilutions, and each dilution was PCR amplified. Results from agarose gel electrophoresis demonstrated a sensitivity of up to 10^−9^, with a detection sensitivity of 0.2 CFU/μL (see Figure 4).

### 3.5 Clinical application and validation of PCR in diagnostic settings

Samples of cow feces and nasal secretions were collected from dairy farms across five provinces in China: Shandong, Zhejiang, Jiangxi, Anhui, and Heilongjiang. The PCR method for detecting *M. morganii* showed 100% agreement with conventional detection methods. Among 771 samples tested, 11 were positive, yielding a prevalence of 1.4% (11/771).

## 4 Results of ELISA

### 4.1 Construction and identification of the recombinant plasmid pcDNA3.1-His-LPP

**Figure 5:**
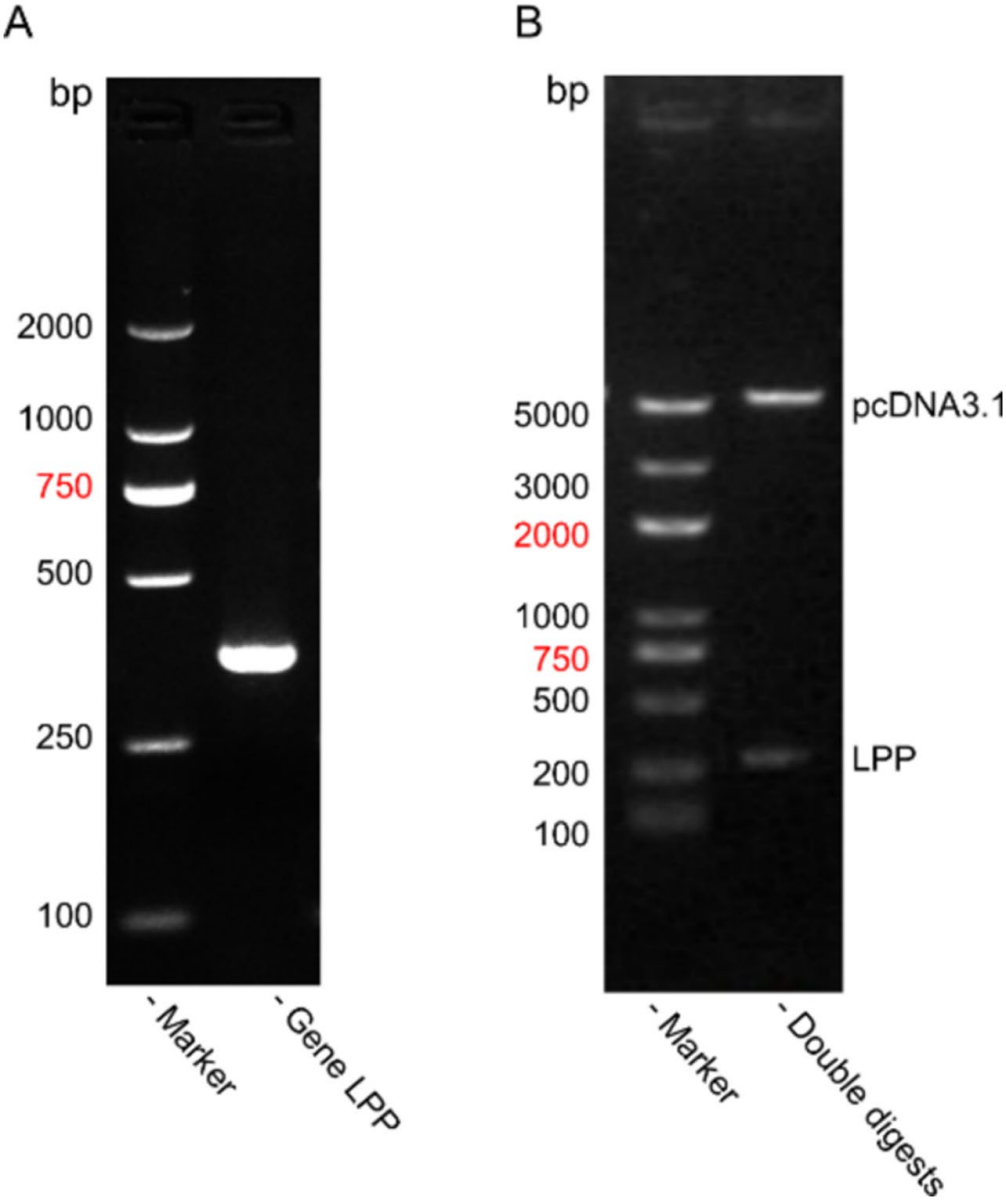
Construction and Identification of the recombinant plasmid pcDNA3.1-His-LPP. **(A)** Result of PCR in recombinant plasmid pcDNA3.1-His-LPP. **(B)** Identification of the recombinant plasmid pcDNA3.1-His-LPP.

Agarose gel electrophoresis analysis confirmed successful PCR amplification of a 276 bp LPP protein gene, which matched the expected band size (Figure 5A). Following the construction of the recombinant plasmid, subsequent electrophoresis revealed fragments of 276 bp and 5,158 bp after double digestion, consistent with the anticipated sizes (Figure 5B).

### 4.2 Expression and purification of recombinant LPP protein

**Figure 6:**
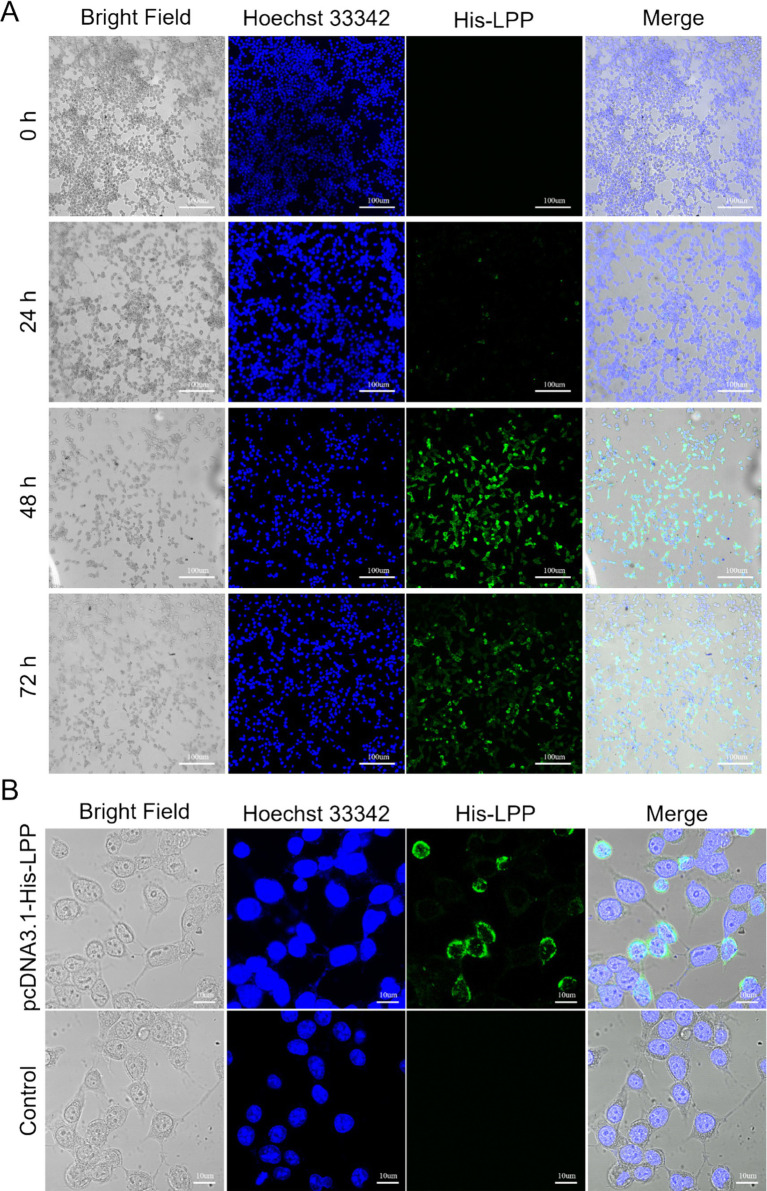
Expression detection of LPP protein. **(A)** Images of HEK293T cells under a fluorescence microscope, with Hoechst 33342 staining used to label nuclei (blue) and LPP protein expression (green) observed at 0, 24, 48, and 72 h. Scale bar: 100 μm. **(B)** Detection of LPP protein expression forms. Scale bar: 10 μm.

**Figure 7:**
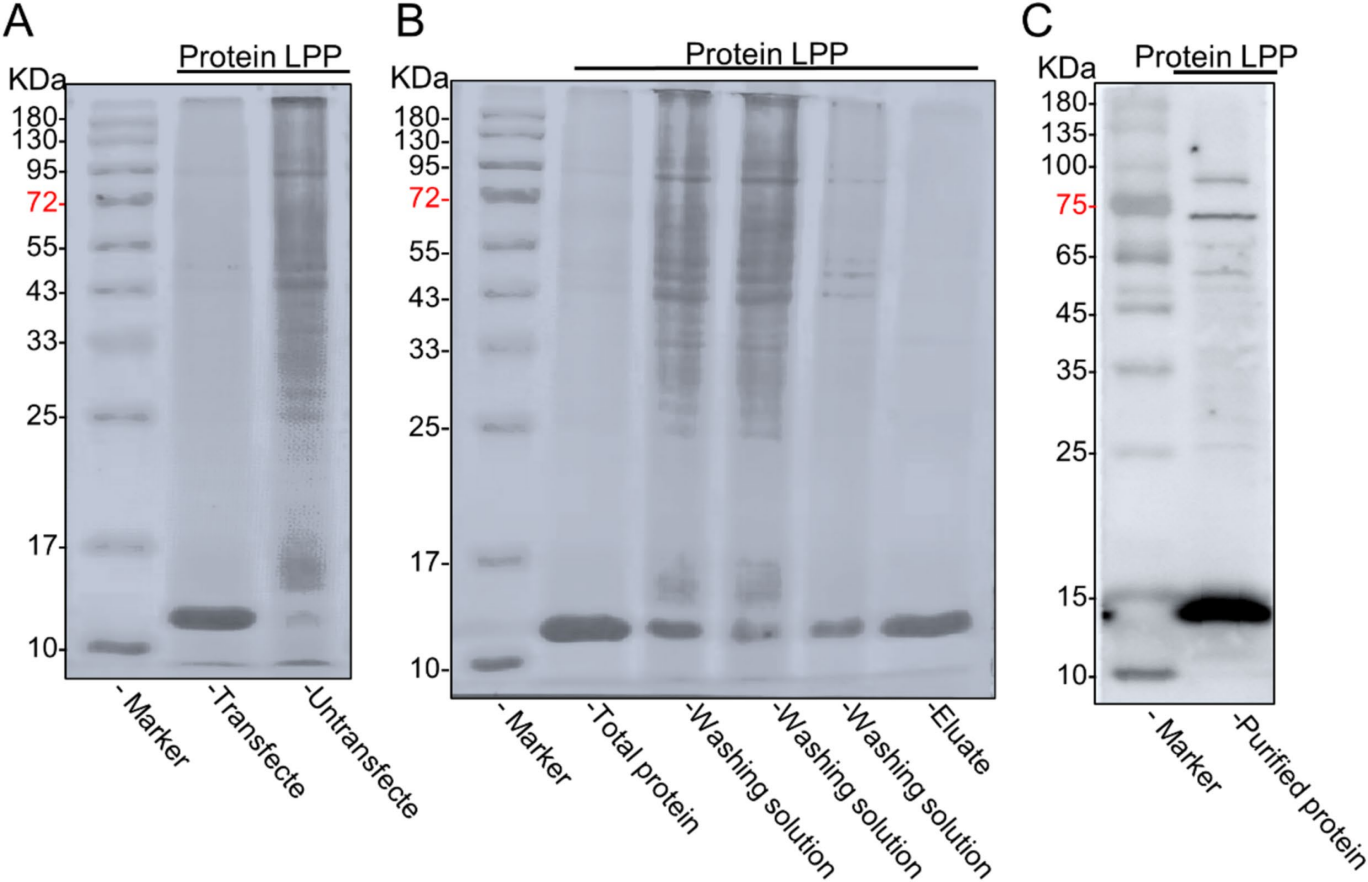
Expression and purification of LPP recombinant protein. **(A)** Verification of LPP protein expression. The cells transfected with the plasmid expressed the protein (lane 1), while the cells without the plasmid did not express the protein (lane 2). **(B)** SDS-PAGE of the LPP protein from 293T cells expressed recombinant LPP protein (lane1), penetration fluid (lane 2), wash fractions with imidazole (lanes 3-4), and purified recombinant LPP protein eluted with imidazole (lane 5), with strip-out fraction (lane 8) and molecular weight markers: 10, 17, 25, 33, 43, 55, 72, 95, 130, and 180 kDa (lane M). **(C)** The Western blot analysis was conducted to verify the purified protein.

The DNA encoding the LPP protein was cloned into the pcDNA3.1(+) expression vector, resulting in the plasmid pcDNA3.1(+)—LPP. The expression of pcDNA3.1(+)—LPP was assessed in 293 T cells at various time points (0, 24, 48, and 72 h), with peak yield observed at 48 h (Figure 6A). Under optimized culture conditions, the protein was observed to be secreted in a soluble form within the cytoplasm of the cells via inverted fluorescence microscopy (Figure 6B). SDS-PAGE results further confirmed successful protein expression, showing a distinct band at 12 kDa (Figure 7A). Purified target protein also exhibited a clear band at 12 kDa with no background, indicating successful extraction and high purity (Figure 7B). Western blot analysis using an anti-His antibody revealed a clear band at 12 kDa (Figure 7C).

### 4.3 Detection results of polyclonal antibody titer

**Figure 8:**
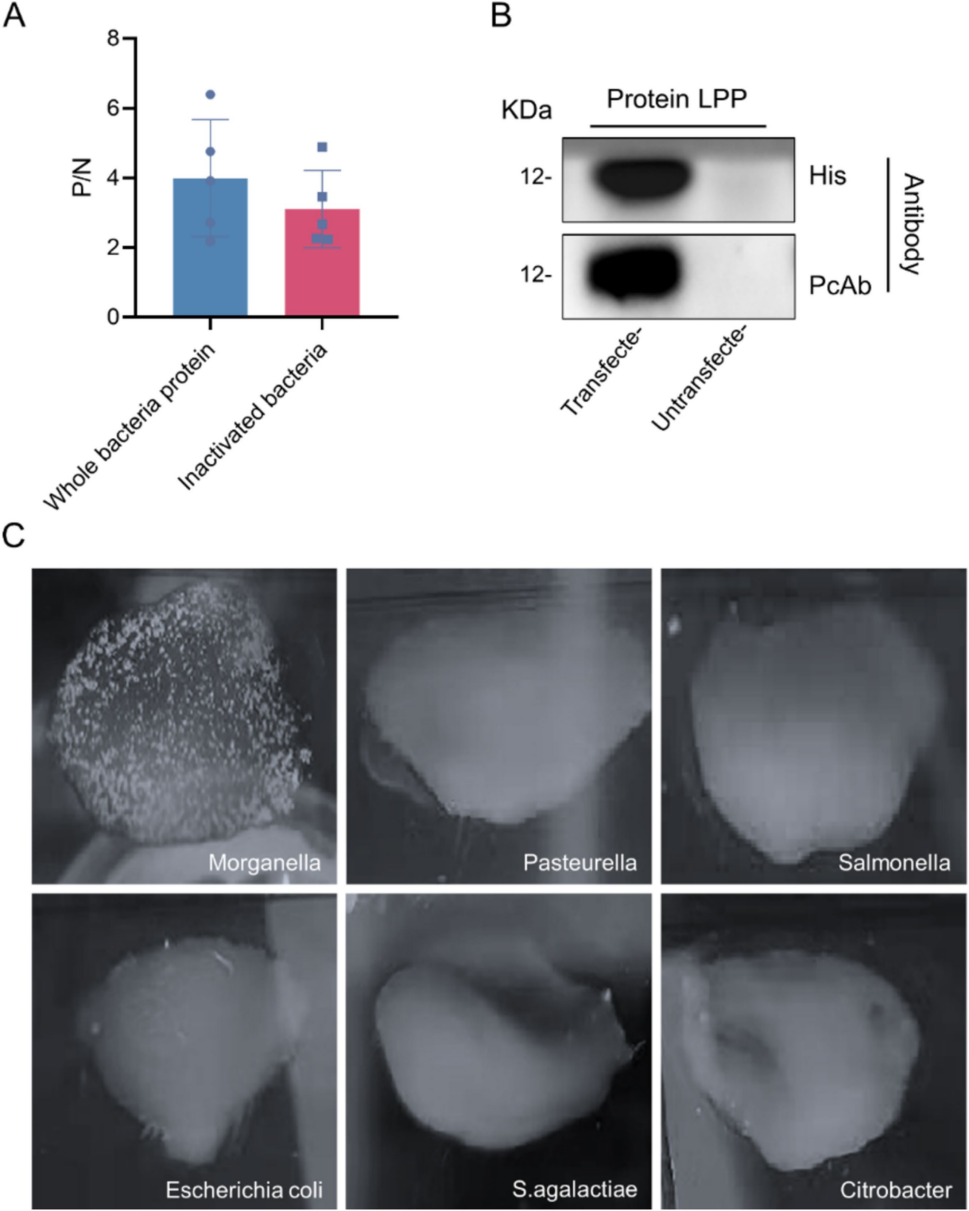
Polyclonal antibody test results. **(A)** Detection of polyclonal antibody titer. **(B)** Western blot analysis of LPP protein. The LPP (12 kDa) probed with *M. morganii* positive sera and the LPP (12 kDa) probed with anti-HIS antibody. **(C)** Polyclonal antibody specificity detection.

After completing the immunization protocol, mouse tails were clipped to collect blood samples, which were then analyzed for antibody titers using indirect ELISA. The results indicated that sera from mice immunized with whole *M. morganii* protein and inactivated *M. morganii* achieved titers exceeding 1:12,800, with a maximum of 1:204,800 (Figure 8A), demonstrating a high antibody level suitable for subsequent experiments. In western blot analyses, the polyclonal antibody showed good binding to the recombinant LPP protein, with no reaction observed with the empty vector protein (Figure 8B). Additionally, agglutination experiments with various bacteria using the prepared polyclonal antibody showed agglutination only with *M. morganii*, confirming the specificity of this antibody (Figure 8C).

### 4.4 Development and optimization of the indirect ELISA for LPP detection

**Figure 9:**
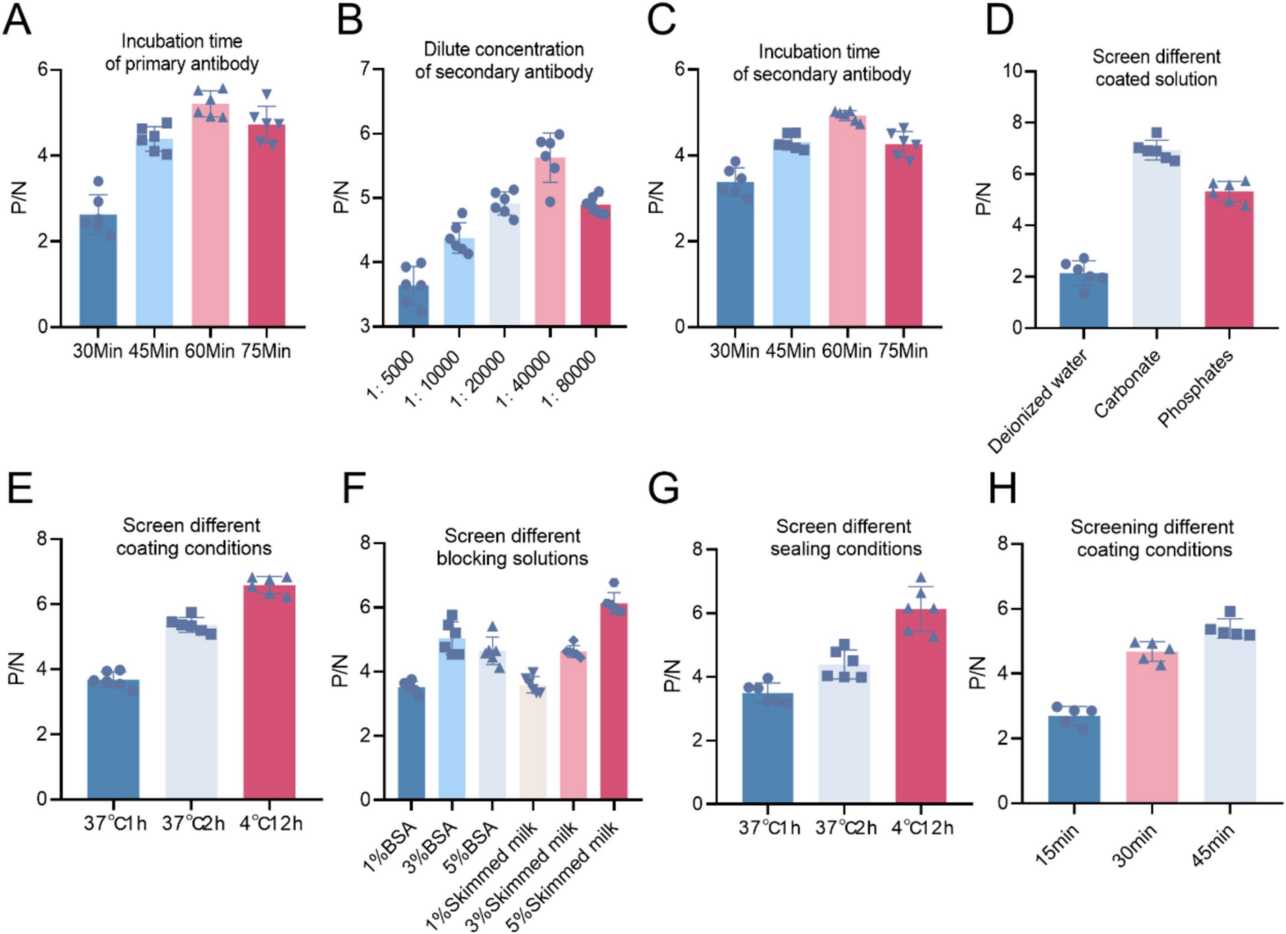
**(A)** Selection of primary antibody incubation time. **(B)** Optimal dilution concentration of the enzyme-labeled secondary antibody. **(C)** Optimal incubation time for the secondary antibody. **(D)** Selection of coating solution for the primary antibody. **(E)** Selection of coating conditions for the primary antibody. **(F)** Selection of blocking solution. **(G)** Selection of blocking conditions. **(H)** Based on the selected conditions and keeping other factors constant, the optimal incubation time for the substrate TMB was determined.

Using a checkerboard titration method, the optimal dilution for recombinant protein and polyclonal antibodies was determined. Results showed that at a coating concentration of 1:1,600 for the protein and a dilution of 1:12,800 for the polyclonal antibodies, the P/N ratio was maximized. Therefore, the optimal coating concentration for the recombinant protein was established at 1:1,600, corresponding to a protein concentration of 2 μg/mL, while the optimal working concentration of the polyclonal antibodies was determined to be 1:12,800 (Table 1). Experimental results indicated that the optimal incubation time for the primary antibody was 60 min, as this yielded the highest P/N ratio (Figure 9A). Similarly, the optimal dilution for the enzyme-conjugated secondary antibody was 1:40,000, which also provided the highest P/N ratio (Figure 9B). Furthermore, the optimal incubation time for the secondary antibody was also determined to be 60 min (Figure 9C). Additionally, the results indicated that when carbonate buffer was used as the coating solution, the P/N ratio reached its peak, establishing carbonate as the optimal coating solution (Figure 9D). Likewise, a coating condition of 4°C for 12 h yielded the highest P/N ratio, indicating this is the optimal coating condition (Figure 9E). Regarding blocking solution selection, using 5% non-fat dry milk resulted in the highest P/N ratio, thereby establishing it as the optimal blocking solution (Figure 9F). Moreover, a blocking condition of 4°C for 12 h also resulted in the highest P/N ratio, confirming it as the optimal blocking condition (Figure 9G). Based on these selected conditions, substrate TMB was added while maintaining other conditions constant, with substrate incubation times chosen as 15 min, 30 min, and 45 min at room temperature. Results demonstrated that the optimal substrate incubation time was 45 min, yielding the highest P/N ratio (Figure 9H).

**Table 1:** Negative serum OD_450_ nm values.

OD_450_ nm value					
0.084	0.089	0.086	0.093	0.055	0.058
0.076	0.058	0.077	0.099	0.094	0.057
0.083	0.079	0.079	0.098	0.065	0.055
0.058	0.07	0.076	0.074	0.084	0.054
0.061	0.095	0.100	0.064	0.076	0.053
0.072	0.091	0.081	0.062	0.082	0.059

### 4.5 Determination of cut-off values for indirect ELISA

Based on the established optimal conditions for coating the ELISA plates, a total of 36 negative serum samples were tested to determine the cut-off values. The average OD_450_ was calculated to be 0.075, with a standard deviation (SD) of 0.0147. According to statistical principles, a serum sample with an OD_450_ nm value greater than 0.119 was classified as positive, while a value less than 0.104 was classified as negative, with values in between considered suspect (Table 1).

### 4.6 Specificity of the indirect ELISA method

**Figure 10:**
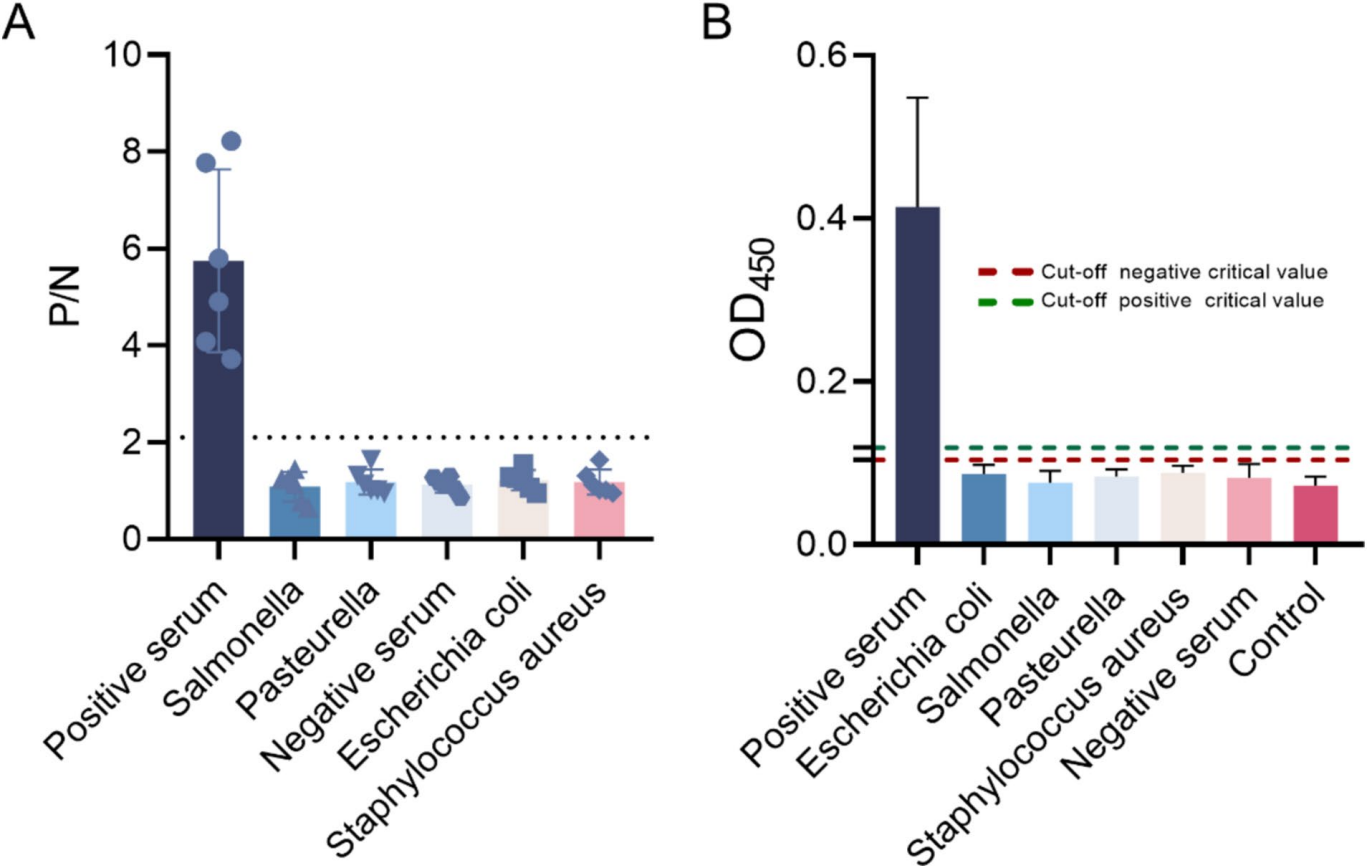
Specific detection using the indirect ELISA method. **(A)** The P/N values and **(B)** cut-off values of the negative sera, *Escherichia coli*, *Salmonella*, *Pasteurella* spp., *Staphylococcus aureus*, and negative sera were evaluated, confirming the specificity of the method.

The established method was applied to detect various pathogenic sera, including *Salmonella*, *Bacillus subtilis*, *Staphylococcus aureus*, *Escherichia coli*, and *Pasteurella* spp. The results were assessed based on the P/N ratio and cut-off values, indicating that the indirect ELISA method developed does not exhibit cross-reactivity with the aforementioned pathogens (Figure 10).

### 4.7 Repeatability and stability of indirect ELISA assays

Twelve serum samples positive for *M. morganii* and 12 negative samples were selected to evaluate the reproducibility of the indirect ELISA method using both intra- and inter-batch testing. Statistical analysis of the results indicated that the within-batch coefficient of variation (CV) ranged from 0.6 to 13.2%, while the inter-batch CV ranged from 0.6 to 10.3%, less than 15%. These results demonstrate the good reproducibility and stability of the method (Tables 2, 3).

**Table 2:** Within-batch coefficient of variation.

Sample number	OD value			Average value ( $\bar{X}$ )	Standard deviation (SD)	Coefficient of variation (CV) (%)
	1	2	3			
1	1.046	1.07	1.09	1.069	0.018	1.683
2	1.327	1.345	1.329	1.334	0.008	0.604
3	0.849	0.857	0.796	0.834	0.027	3.246
4	0.606	0.659	0.621	0.629	0.022	3.548
5	1.74	1.691	1.792	1.741	0.041	2.369
6	1.667	1.652	1.649	1.656	0.008	0.475
7	1.617	1.692	1.658	1.656	0.031	1.852
8	1.316	1.423	1.443	1.394	0.056	4.000
9	1.773	1.758	1.737	1.756	0.015	0.841
10	0.959	0.925	0.934	0.939	0.014	1.531
11	1.07	1.119	1.103	1.097	0.020	1.859
12	1.142	1.129	1.109	1.127	0.014	1.205
13	0.075	0.072	0.076	0.074	0.002	2.287
14	0.072	0.072	0.07	0.071	0.001	1.322
15	0.066	0.069	0.068	0.068	0.001	1.843
16	0.061	0.062	0.059	0.061	0.001	2.056
17	0.089	0.084	0.083	0.085	0.003	3.076
18	0.067	0.061	0.066	0.065	0.003	4.059
19	0.07	0.071	0.077	0.073	0.003	4.254
20	0.055	0.05	0.059	0.055	0.004	6.735
21	0.045	0.044	0.041	0.043	0.002	3.922
22	0.079	0.073	0.073	0.075	0.003	3.771
23	0.046	0.043	0.058	0.049	0.006	13.226
24	0.052	0.053	0.049	0.051	0.002	3.311

**Table 3:** Inter-batch coefficient of variation.

Sample number	OD value			Average value ( $\bar{X}$ )	Standard deviation (SD)	Coefficient of variation (CV) (%)
	Plate 1	Plate 2	Plate 3			
1	1.092	1.022	1.057	0.793	0.029	3.605
2	1.269	1.283	1.272	0.956	0.006	0.630
3	0.905	0.925	0.901	0.683	0.010	1.538
4	0.719	0.697	0.829	0.561	0.058	10.288
5	1.768	1.788	1.791	1.337	0.010	0.764
6	1.639	1.782	1.685	1.277	0.060	4.669
7	1.684	1.669	1.642	0.877	0.010	1.171
8	1.339	1.301	1.388	1.007	0.036	3.536
9	1.828	1.822	1.918	0.656	0.030	4.601
10	0.895	0.832	0.897	1.249	0.017	1.392
11	1.158	1.183	1.168	1.392	0.044	3.154
12	1.118	1.166	1.173	0.864	0.024	2.828
13	0.079	0.088	0.085	0.063	0.004	5.939
14	0.060	0.063	0.063	0.047	0.001	3.041
15	0.084	0.080	0.081	0.061	0.002	2.775
16	0.074	0.074	0.071	0.055	0.001	2.583
17	0.089	0.098	0.093	0.070	0.004	5.260
18	0.074	0.064	0.067	0.051	0.004	8.175
19	0.075	0.072	0.069	0.054	0.002	4.536
20	0.061	0.056	0.061	0.045	0.002	5.297
21	0.052	0.047	0.053	0.038	0.003	6.907
22	0.084	0.081	0.086	0.063	0.002	3.275
23	0.051	0.053	0.048	0.038	0.002	5.407
24	0.048	0.052	0.056	0.039	0.003	8.367

### 4.8 Concordance analysis of the indirect ELISA with other detection methods

An indirect ELISA test was conducted on 50 bovine serum samples stored in the laboratory. Results showed that the indirect ELISA method detected 10 positive samples and 37 negative samples, while the PCR method detected 11 positive samples and 39 negative samples. The positive concordance rate of this detection method was 90.9% (10/11), and the negative concordance rate was 94.9% (37/39) (Table 4).

**Table 4:** Concordance experiment of indirect ELISA.

	Positive	Negative
	11	39
I-ELISA	10	37
Concordance rate	90.9%	94.9%

### 4.9 Clinical application and validation of the indirect ELISA for *Morganella* detection

A total of 476 serum samples were collected and tested using the indirect ELISA method in dairy farms across 12 cities in Shandong Province, including Jining, Yantai, Weifang, Zaozhuang, Tai’an, Linyi, Dezhou, Dongying, Liaocheng, Zibo, Heze, and Jinan. The results indicated that 28 samples were positive, accounting for 5.9% of the total (Table 5).

**Table 5:** *M. morganii* antibodies in serum samples from farms were classified as positive or negative for *M. morganii*, as determined using the I-ELISA.

Sera source	Positive (copies)	Negative (copies)	Total (copies)	Positive rate (%)
Jining	4	36	40	10
Yantai	5	95	100	5
Weifang	1	19	20	5
Zaozhuang	0	20	20	0
Tai’an	5	35	40	12.5
Linyi	1	59	60	1.67
Dezhou	1	39	40	2.5
Dongying	2	18	20	10
Liaocheng	1	15	16	6.25
Zibo	2	38	40	5
Heze	6	54	60	10
Jinan	0	20	20	0
Total	28	448	476	5.9

## 5 Discussion

*M. morganii* are opportunistic pathogens that rarely cause infections, yet they are implicated in a diverse array of diseases, including sepsis, abscesses, urinary tract infections, cellulitis, diarrhea, and bacteremia. These bacteria are particularly likely to infect newborns and vulnerable individuals in healthcare settings, and infections carry a high mortality rate (26). Since *M. morganii* was first identified, it has continuously appeared in nosocomial infections, with certain strains showing increasing virulence and antibiotic resistance, which has led to higher associated mortality rates (14, 27). The development of improved bacterial detection methods, including rapid detection techniques for *M. morganii*, is increasingly essential. However, detection methods for *M. morganii* have not been developed.

Currently, research on *M. morganii* remains limited. Bovine farming, a rapidly growing industry, plays an important role in the economy, and establishing diagnostic methods for detecting *M. morganii* in cattle is beneficial for the livestock sector, providing a basis for diagnosing and preventing infections. *M. morganii* are primarily found in the feces, urine, and wounds of humans and animals, making PCR detection challenging due to numerous interfering substances in these environments. PCR technology, known for its high sensitivity, specificity, and rapid processing time, is widely used in detecting various pathogens. However, traditional PCR methods are time-intensive due to complex DNA extraction and purification processes, which often lead to extraction failures. Traditional biochemical identification methods are slow, while gene sequencing is both time-consuming and complex (28).

Direct PCR detection from bacterial suspensions has gained attention, but it often encounters interference from contaminants that can result in false-positive results, limiting its application. In response, this study developed a novel direct PCR detection method for *M. morganii*, addressing the limitations of existing direct PCR approaches and providing a new detection strategy. To effectively extract *M. morganii* from samples, the sample was diluted with sterile water, filtered, and then centrifuged at 3,000 rpm for 5 min. After centrifugation, the supernatant was discarded, and the pellet was resuspended in a sterile PBS buffer.

Additionally, the OD value of the bacterial suspension prepared in PBS affects subsequent PCR amplification and electrophoresis outcomes. If the OD is too low, the template quantity is insufficient, and bands on electrophoresis may be faint; if too high, PCR inhibition can occur, resulting in smeared bands. It was determined that an OD value of 0.1–0.15 in the suspension yields high PCR efficiency and clear electrophoresis bands.

Primer design is critical for the effectiveness of direct PCR detection. Unlike conventional PCR, this method requires high precision in primer design. In the experiment, both target regions and PCR conditions were optimized. Traditional direct PCR methods typically require boiling the bacterial suspension prior to amplification, a step that was omitted here. To address possible incomplete denaturation, the pre-denaturation time was extended. After extensive trials, a pre-denaturation time of 5 min was chosen, balancing effective denaturation and polymerase activity. Additionally, since the primers are relatively short and low in GC content, a lower annealing temperature of 50°C significantly improved amplification yield.

Bacterial lipoprotein, a lipid-modified membrane protein, is a vital component of Gram-negative outer membranes and Gram-positive cell envelopes, essential for bacterial survival and pathogenicity. Lipoprotein (LPP) has been identified as a pathogenic factor in many bacteria. The DNA sequence of the lipoprotein gene LPP in *M. morganii* has been determined. This protein’s highly conserved structure, stability in *M. morganii*, and low mutation rate make it a prime candidate for developing an ELISA detection method. Results were consistent with expectations, as optimized ELISA detected positive sera of *E. coli*, *Salmonella*, *Pasteurella*, and *S. aureus* without cross-reactivity with non-*M. morganii* pathogens, demonstrating high specificity and stability.

In this study, a detection method was established using *M. morganii* for both antigen and antibody detection. The PCR method, which targets antigen detection, is highly specific, simple, rapid, and requires low sample purity. The ELISA method, designed for antibody detection, is specific, easy to operate, and suitable for large-scale clinical testing. Both methods have their advantages, allowing for flexible application depending on the situation and laying a foundation for the early clinical diagnosis of *M. morganii*.

## Data Availability

The original contributions presented in the study are included in the article/supplementary material, further inquiries can be directed to the corresponding authors.
